# Design and Preclinical Development of a Phage Product for the Treatment of Antibiotic-Resistant *Staphylococcus aureus* Infections

**DOI:** 10.3390/v11010088

**Published:** 2019-01-21

**Authors:** Susan M. Lehman, Gillian Mearns, Deborah Rankin, Robert A. Cole, Frenk Smrekar, Steven D. Branston, Sandra Morales

**Affiliations:** 1 AmpliPhi Biosciences, San Diego, CA 92130, USA; sml@ampliphibio.com; 2 AmpliPhi Australia, Sydney, NSW 2100, Australia; gm@ampliphibio.com (G.M.); dar@ampliphibio.com (D.R.); rac@ampliphibio.com (R.A.C.); sb@ampliphibio.com (S.D.B.); 3 AmpliPhi d.o.o., 1261 Ljubljana-Dobrunje, Slovenia; frenk.smrekar@jafral.com

**Keywords:** bacteriophage, phage therapy, *Staphylococcus aureus*, biofilm, antimicrobial, frequency of resistance, phage sensitivity, resistance management, nontraditional antibacterial

## Abstract

Bacteriophages, viruses that only kill specific bacteria, are receiving substantial attention as nontraditional antibacterial agents that may help alleviate the growing antibiotic resistance problem in medicine. We describe the design and preclinical development of AB-SA01, a fixed-composition bacteriophage product intended to treat *Staphylococcus aureus* infections. AB-SA01 contains three naturally occurring, obligately lytic myoviruses related to *Staphylococcus* phage K. AB-SA01 component phages have been sequenced and contain no identifiable bacterial virulence or antibiotic resistance genes. In vitro, AB-SA01 killed 94.5% of 401 clinical *Staphylococcus aureus* isolates, including methicillin-resistant and vancomycin-intermediate ones for a total of 95% of the 205 known multidrug-resistant isolates. The spontaneous frequency of resistance to AB-SA01 was ≤3 × 10^−9^, and resistance emerging to one component phage could be complemented by the activity of another component phage. In both neutropenic and immunocompetent mouse models of acute pneumonia, AB-SA01 reduced lung *S. aureus* populations equivalently to vancomycin. Overall, the inherent characteristics of AB-SA01 component phages meet regulatory and generally accepted criteria for human use, and the preclinical data presented here have supported production under good manufacturing practices and phase 1 clinical studies with AB-SA01.

## 1. Introduction

The use of bacteriophages (phages) as antibacterial drugs, frequently referred to as “phage therapy” has been discussed and deployed since these bacterial viruses were discovered in the early 1900s. Interest in phage therapy has waxed and waned in various parts of the world, heavily influenced by the availability, affordability, and efficacy of potent small-molecule antibiotics [1]. The current resurgence of interest is persisting in light of the growing urgency of the antimicrobial resistance (AMR) crisis, which predicts that AMR will be the leading cause of human death by 2050, causing 10 million global deaths per year [2].

*Staphylococcus aureus*, one of the ESKAPE pathogens (*Enterococcus faecium*, *Staphylococcus aureus*, *Klebsiella pneumoniae*, *Acinetobacter baumannii*, *Pseudomonas aeruginosa*, and *Enterobacter spp*.) [3], is a problem in both hospital-associated and community-associated infections [4,5]. It is a significant problem in many clinical settings and the antibiotic-resistant forms are classified as a “High Priority” pathogen by the World Health Organization (WHO) [6] and a “Serious Threat” by the U.S. Centers for Disease Control and Prevention (CDC) [7]. Since 1999, nine antibiotics targeting methicillin-resistant *S. aureus* (MRSA) have been approved (linezolid, daptomycin, tigecycline, ceftobiprole, telavancin, ceftaroline, dalbavancin, oritavancin, tedizolid) [8]. Of these, only the oxazolidinones, now nearly 20 years old, were a completely new class [9]. Clinical resistance has already been observed for all nine of these drugs, though it can be difficult to predict how quickly or widely some of these resistances will spread [10,11,12,13,14,15,16,17,18,19]. Side effects such as renal toxicity and cross-resistance (e.g. among glyo- and lipoglycopeptides) can limit clinical use [8]. Moreover, many antibiotics have reduced efficacy against *Staphylococcus* spp. when it grows in biofilms [20], as is often the case with device-associated infections or endocarditis. Thus, there remains an urgent need for anti-staphylococcal drugs, especially ones with fundamentally different mechanisms of action. Here, we describe the design and composition of AB-SA01, a highly characterized anti-*Staphylococcus* phage product that is being developed to treat acute and chronic *S. aureus* infections in humans, including those caused by MRSA.

Phage therapy has frequently been cited as a form of technology that could help address the AMR problem, provided that high-quality evidence can be gathered in controlled studies focused on testing product efficacy in well-defined clinical indications and administration parameters [7,21,22,23]. Phages are unable to infect mammalian cells and are usually specific for one or a few bacterial species or strains. Obligately lytic phages would comprise a self-replicating, self-limiting antimicrobial that can be administered by a variety of routes, and that functions via an entirely different mechanism of action compared to small-molecule antibiotics. Humans are continuously exposed to phages present in the environment and as part of the human microbiome, and there is no evidence of any direct toxicity resulting from intentionally administered phages as long as non-phage contaminants such as endotoxins are removed [24,25,26].

While there remains some debate about the optimal features of therapeutic phages, there is widespread agreement that the traits listed below are either required or particularly desirable for phage products and their individual components [27,28,29,30,31]. Individual phages should be:

- Obligately lytic, to avoid specialized transduction of bacterial genes, and maximize chances for bacterial killing;
- Not known, by empirical testing and/or inference from genomics, to be prone to generalized transduction; and,
- Fully sequenced, to avoid phages with known antibiotic resistance or bacterial virulence genes, and to help assess other lifestyle traits.

Collectively, phages used together to treat a patient should:

- Have broad activity against the target pathogen but not other species, to maximize potential utility and minimize off-target effects; and,
- Be capable of complementation, in which resistant mutants arising to one phage are sensitive to another phage.

In addition to characteristics of the phages themselves, material for clinical use should be produced in such a way as to give confidence that the final product retains these characteristics (i.e., are still the same phages) and does not contain potentially harmful (or harmful amounts) of impurities such as endotoxin or host cell proteins. AB-SA01 satisfies these criteria and has entered clinical development.

## 2. Methods

### 2.1. Bacteriophages, Source and Propagation

Each of the selected phages was isolated from an environmental source and subsequently paired to a well-characterized *S. aureus* strain that serves as its manufacturing host. Host-paired phages were purified to ensure that the resulting master stocks produced genetically and phenotypically consistent batches of each phage. Unless otherwise stated, all data is derived from the host-paired, plaque-purified phages. Phages were propagated in liquid culture using vegetable peptone media (VP0101, Oxoid, Hampshire, UK). Lysates were passed through a 0.2-µm filter to remove large cellular debris and, depending on the needs of subsequent testing, optionally subjected to a proprietary process of column-based purification steps to further remove host cell proteins and other bacterial debris and to replace growth medium with phosphate-buffered saline (PBS; Oxoid, Hampshire, UK) containing 10 mM magnesium sulfate (PBS+Mg).

### 2.2. Bacteria

AB-SA01 manufacturing hosts are *S. aureus* strains originally isolated from humans. The *S. aureus* diversity panel and the species-specificity panel were sourced from the American Type Culture Collection (Manassas, VA, USA), the Walter Reed Army Institute of Research Multidrug-resistant Organism Repository and Surveillance Network (“MRSN”, Silver Spring, MD, USA), and clinical sites in Australia and the United Kingdom. Global surveillance panels of *S. aureus* strains were obtained from JMI Laboratories (North Liberty, IA, USA). Targeted interest panels included chronic rhinosinusitis (CRS) strains from Belgium, and vancomycin intermediate (VISA) strains from the CDC and Food and Drug Administration (FDA) Antimicrobial Resistance Isolate Bank (Atlanta, GA, USA). The definition of multidrug resistant (MDR) strains is according to Magiorakos et al [32].

### 2.3. Phage Sensitivity Assays

Testing on the *S. aureus* panels used Heart Infusion Broth (BD, Franklin Lakes, NJ, USA), amended with 1.5% agar (Oxoid, Hampshire, UK) for plates or 0.7% agar for overlays. Phage activity was assessed using a modification of the small drop agar overlay method [33]. Briefly, 100 µL of 16–18 h planktonic bacterial culture was mixed with molten 0.7% top agar and poured evenly over an agar plate. When the top agar layer was set, serial dilutions of standardized phage solutions were spotted onto the overlay and plates incubated overnight at 37 °C. Phage activity was indicated by clearing of the bacterial lawn at the site of phage application, and by the development of individual plaques as the phage sample is diluted. Strains were only considered sensitive if discrete plaques could be observed as the sample was diluted, indicating phage replication. The titer for each phage+bacteria combination tested was calculated from the drop dilutions. Testing on the species-specificity panel was conducted similarly, using media recommended for the specific bacterial species and bacterial culture volumes suitable to produce a uniform lawn.

### 2.4. Frequency of Resistance and Complementation

Complementation studies conducted during product selection used apparent bacteriophage-insensitive mutant (BIM) colonies that were isolated after infecting a sensitive *S. aureus* strain with the individual candidate phages. Surviving colonies were streak-purified once on agar plates. The double-drop method was then used to screen for phage sensitivity: 10 µL spots of PBS or phage (~1 × 10^9^ plaque-forming units (PFU)/mL) were spotted onto nutrient agar plates and after 10 min, 5 µL of overnight nutrient broth culture from each BIM or the parental strain was applied to each phage spot. After 24 h incubation at 37 °C, phage+bacteria spots were compared to PBS+bacteria controls and scored as R (resistant, no difference from control spot), I (intermediate, phage activity seen within bacterial spot), or S (sensitive, <10 bacterial colonies in spot).

For the final AB-SA01 composition, the frequency of spontaneous phage resistance in triplicate populations of the same *S. aureus* strain was assessed using a modification of the method of O’Flynn et al. [34]. In a final volume of 200 µL, 6–8 × 10^8^ colony forming units (CFU) in nutrient broth was mixed with 2–3 × 10^9^ PFU of purified phage (AB-SA01 or individual components), incubated for 10 min at 37 °C, then mixed with 3 mL molten 0.4% nutrient agar and poured over a 90-mm round 1.5% nutrient agar plate. Bacterial colonies were counted after 24 h and 48 h of incubation at 37 °C. The apparent frequency of BIMs was calculated as the number of colonies on each test plate divided by the input number of bacteria in that replicate. Results were compared using a repeated measures ANOVA and a priori planned comparisons between AB-SA01 and the three component phages were conducted using paired *t*-tests. Up to 10 BIMs from each phage+host combination (all BIMs if <10) were picked and streak-purification was attempted on agar plates.

### 2.5. Genome Sequencing and Analysis

Phage genomic DNA was purified from filtered lysates or purified preparations and sequenced by Illumina paired-end (ACGT, Wheeling, IL, USA) or PacBio technologies (Expression Analysis, Durham, NC, USA), using PCR-free libraries (Illumina TruSeq PCR-free Library Prep kit, PacBio SMRTbell library). Nucleotide sequences have been deposited in GenBank (Sa83: MK417514, Sa87: MK417515, J-Sa36: MK417516). Annotation was conducted using myRAST v36 (http://blog.theseed.org/servers/). Similarities of (1) annotated proteins to all *Staphylococcus* integrases in GenBank and (2) annotated genes to a proprietary database of bacterial virulence and antibiotic resistance genes were assessed using BLAST searches requiring at least 30% identity across 50% of the sequence, and E ≤ 0.05; any hits were manually inspected for validity based on factors such as the likely accuracy of the hit’s original annotation and evidence from secondary structure predicted by HHPred (https://toolkit.tuebingen.mpg.de) [35,36]. Genome alignments were constructed using Progressive Mauve (http://darlinglab.org/mauve/mauve.html) with the default parameters [37].

### 2.6. Animal Studies

Purified phage material was used for all animal studies. **(1) Prototype 4-phage product:** Six groups of five female CD-1 mice (Harlan Laboratories, Houston, TX, USA) were rendered neutropenic by administering 150 and 100 mg/kg cyclophosphamide on day -4 and day -1 prior to infection, respectively. Mice were anaesthetized by intraperitoneal (IP) injection of 0.15 mL of a mixture of ketamine HCl (100 mg/kg body weight) plus xylazine (10 mg/kg body weight). Once anaesthetized, an inoculum of 9.5 × 10^6^ CFU of MRSA strain UNT144-3 was delivered intranasally (IN) in a 50 µL volume. At 2 and 6 hours post-infection (hpi), untreated controls received 50 µL PBS+Mg IN, antibiotic controls received 100 mg/kg vancomycin as a subcutaneous (SC) injection, and the three phage treatment groups received 1 × 10^9^ PFU per phage, 1 × 10^8^ PFU per phage, or 1 × 10^7^ PFU per phage in a 50-µL IN dose. At 24 hpi, mice were euthanized by CO_2_ inhalation and lungs processed for bacterial load. Bacterial counts were enumerated on Brain Heart Infusion agar with 0.5% activated charcoal. **(2) AB-SA01:** Three groups of five female BALB/c mice were anesthetized and an inoculum of 3.0 × 10^8^ CFU of methicillin-sensitive *S. aureus* strain Xen29 was delivered IN in a volume of 35 µL. At 2 and 6 hpi, untreated controls received 50 µL PBS-Mg IN and the phage treatment group received 5 × 10^8^ PFU per phage in a 50 µL IN dose. At 2, 6, and 12 hpi, the antibiotic controls received 110 mg/kg vancomycin as a SC injection. At 24 hpi, mice were euthanized by CO_2_ inhalation and lungs processed for bacterial load. Care was taken to ensure tissue samples were kept cold and processed promptly for bacterial presence. Bacterial counts were enumerated on Mueller Hinton agar. After both mouse studies, bacteria recovered from mouse lung tissue were tested for phage sensitivity according to the method of 2.3. **Statistical Analysis:** Treatments were compared by one-way ANOVA on log_10_-transformed values with Tukey’s test for all pairwise comparisons. Adjusted *p*-values are reported. **Bacterial strains:** *S. aureus* strains were provided by the vendors conducting the studies. UNT144-3 is MRSA and carries the *tetM* gene. Xen29 [38] is available from Perkin Elmer, Inc. Media choice for bacterial enumeration was per each vendor’s standard practice. Each vendor had previously established both the dosing for their vancomycin control groups and the bacterial inoculation methods yielding consistent infection outcomes for each specific mouse and *S. aureus* strain combination.

### 2.7. Animal Welfare

Mouse studies were conducted by external vendors. Study “AmpliPhi 2014-01”: The University of North Texas Health Science Center Animal Facility is a member in good standing with the Association for Assessment and Accreditation of Laboratory Animal Care International. Study “APP004-2” (study approved21 March, 2016): KWS BioTest conducts all in-life experimental procedures in accordance with United Kingdom Animals (Scientific Procedures) Act 1986. Their local Ethical Review Process occurred under the auspices of the University of Bristol’s Animal Welfare and Ethical Review Body (AWERB).

## 3. Results

### 3.1. Physicochemical Characteristics of AB-SA01 Component Phages

**Figure 1:**
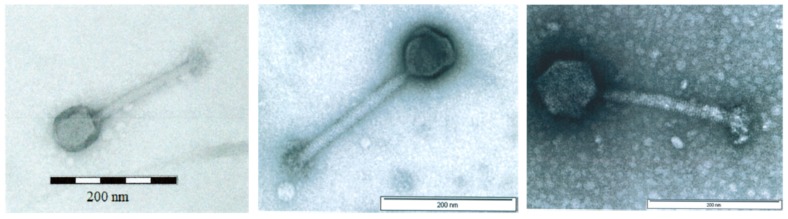
Transmission electron microscopy images of (left to right) Sa83, Sa87, and J-Sa36. Scale bars: 200 nm. Filtered lysates were PEG8000 precipitated, suspended in salt-magnesium buffer, stained with 2% uranyl acetate, and imaged at 80–100kV [39].

All three AB-SA01 component phages produce small, clear plaques when plated on their paired *S. aureus* hosts. Transmission electron microscopy (TEM) images of the three AB-SA01 component phages show the straight, contractile tail and narrow neck that are characteristic of phages belonging to the order *Caudovirales*, family *Myoviridae* (Figure 1).

**Figure 2:**
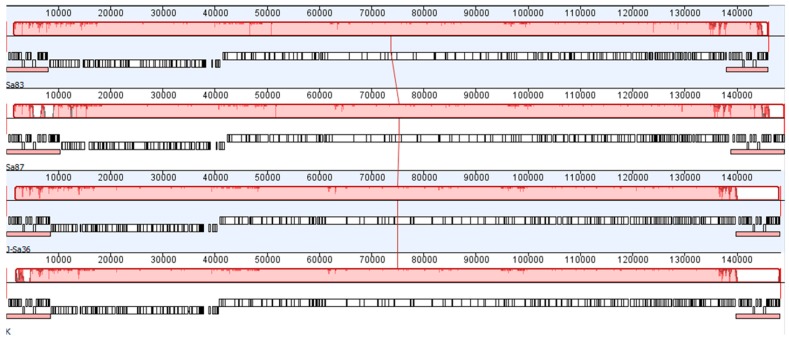
A Progressive Mauve alignment of (top to bottom) Sa83, Sa87, J-Sa36, and phage K (GenBank K766114), each showing annotated genes (white boxes) and long terminal repeats (small red boxes immediately below white gene blocks). The large red blocks above each annotated genome (connected by the red vertical line at approximately 75 kb) represent local collinear blocks of genomes identity; interruptions in these red blocks indicate differences among the four aligned nucleotide sequences.

All AB-SA01 component phages were sequenced from amplification-free libraries capable of revealing the relative frequencies of genome regions. Read-mapping data showed regions of approximately doubled coverage identifying the genome termini and associated fixed direct terminal repeats between approximately 8 and 10 kb. These genome structures indicate a sequence-specific packaging mechanism not associated with generalized transduction. The pairwise relatedness of the collinear single-copy component phage genomes ranges from 93 to 97% nucleotide identity (Figure 2) and all are related to well-studied *S. aureus* myovirus phage K. No identifiable integrases were found in the AB-SA01 component phage genomes and none of the ca. 200 predicted phage genes in each of the three phages were similar to known bacterial virulence or antibiotic resistance genes. 

### 3.2. In Vitro Activity of AB-SA01

The target species for AB-SA01 is *S. aureus*. Overall, 94.5% of 401 clinical *S. aureus* isolates were sensitive to AB-SA01 (Table 1), including 95% of the 205 total isolates known to be MDR, and with little apparent variation by genetic lineage (Supplementary Table S1), year of isolation, or infection type. When tested on representatives of normal human microflora and related staphylococci, AB-SA01 and its component phages showed some activity against two of five tested *S. epidermidis* strains, but no cross-genus activity (Table 2). When tested on *S. aureus* strains, no evidence of interference among the component phages was observed. The titers observed for AB-SA01 were mostly consistent with the component phage activities, except for a few cases of apparent synergy in which AB-SA01 generated plaques on the *S. aureus* strain even though none of the individual component phages did so. Since testing was conducted in triplicate, this observation of synergy is likely to be real, as opposed to a case of borderline results in which plaques were a bit more obvious with AB-SA01 by simple chance.

The AB-SA01 component phages were selected partly based on the 68-member diversity panel, which included representatives of all major community-acquired (CA-) and hospital-acquired (HA-) MRSA lineages [4,5]. Each component phage had a different host range, with most bacterial strains being sensitive to more than one of the AB-SA01 phages. AB-SA01 activity was similarly high across panels of isolates that represent globally prevalent *S. aureus* from blood, wound, lung, urinary, and other infections in later years. Using targeted interest panels, AB-SA01 was also shown to have activity on strains being relatively rare but concerning the VISA phenotype, a panel of exclusively CRS isolates, and a variety of the clinically significant USA300 lineage.

### 3.3. Frequency of Resistance and Complementation

The potential for phages to complement each other in the event that bacterial resistance arises was considered as part of AB-SA01 development. During product selection, six candidate phages with broad or differing host ranges were assessed on a sensitive *S. aureus* strain. Using the double-drop method of 2.4, BIMs that were generated using one phage were first tested to confirm whether they truly exhibited reduced phage sensitivity after streak-purification, then cross-resistance to other phages was tested (Table 3). Sa83, Sa81, and Sa76 were similarly able to complement Sa87-induced resistance and had previously shown very similar host ranges. Of these, only Sa83 was retained because it made a slightly better contribution to the total host range and complementation profile of AB-SA01. J-Sa37 more often exhibited cross-resistance than complementation and was not included in AB-SA01. J-Sa36 exhibited different complementation behavior as compared to Sa87 or Sa83.

After AB-SA01 composition was finalized, the mean apparent frequency of resistance to AB-SA01 was lower than the values observed for the individual phages, both at 24 h and 48 h (Table 4). However, this trend was not statistically significant (*p* > 0.05), possibly because the values observed in this study were close to the limit of detection. This suggests that the spontaneous frequency of AB-SA01 resistance among sensitive *S. aureus* populations is no greater than ~3 × 10^−9^. None of the BIM colonies observed in this study could be recovered by picking and re-streaking on agar to isolate them away from phages on the original test plate, implying that their growth on the test plates was not due to stable, heritable phage resistance, but was instead a temporary phenotype or a spatial phenomenon in which cells escape contact with the phages during incubation of the phage-bacteria mixture before plating.

### 3.4. In Vivo Activity of AB-SA01

**Figure 3:**
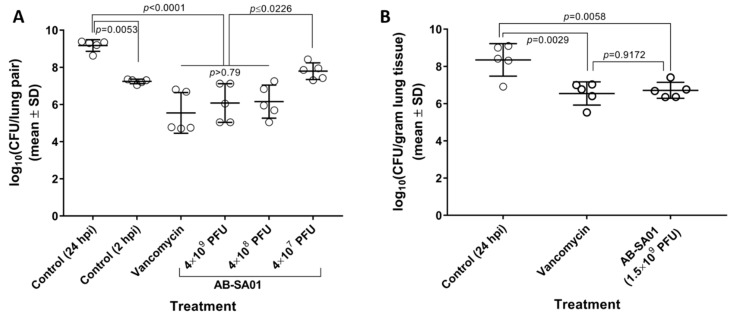
AB-SA01 reduces lung bacterial burden in (**A**) neutropenic CD-1 mice and (**B)** immunocompetent BALB/c mice. Phage doses are given as total plaque-forming units (PFU) per dose.

AB-SA01 showed efficacy equivalent to vancomycin in two murine acute lung infection models, each of which used a different *S. aureus* challenge strain, murine genetic background, and immune status. In the first murine pneumonia model (Figure 3A), three doses of the AB-SA01 prototype were tested in neutropenic CD-1 mice. This prototype contained the three phage components of AB-SA01 plus the J-Sa37 phage that was later removed from the product because its fractional contribution to in vitro host range and complementation were deemed insufficient to justify manufacturing a fourth component phage. At 24 hpi, lung homogenates from mice treated with 4 × 10^9^ or 4 × 10^8^ total PFU contained significantly fewer bacteria than mice treated with buffer and were statistically equivalent (all *p* > 0.79) to mice that had been treated with vancomycin at the same time points. The mean reductions in lung bacterial load relative to untreated mice were 3.63 log_10_CFU (*p* < 0.0001) for the vancomycin group, 3.09 log_10_CFU (*p* < 0.0001) for the highest AB-SA01 dose group, and 3.02 log_10_CFU (*p* < 0.0001) for the medium AB-SA01 dose groups. These results suggested that doses higher than 4 × 10^7^ PFU were required for efficacy in this model.

In a follow-up experiment using the final AB-SA01 composition (Figure 3B), a 1.5 × 10^9^ total PFU dose group was tested in immunocompetent BALB/c mice. At 24 hpi, lung homogenates from mice that had received two doses of AB-SA01 contained statistically fewer bacteria than those from untreated mice (*p* = 0.0058), and were statistically equivalent to mice that had received three doses of vancomycin (*p* = 0.9172). The mean reductions in lung bacterial load relative to the untreated control were 1.64 log_10_CFU for AB-SA01-treated mice and 1.80 log_10_CFU for vancomycin-treated mice.

In both mouse studies, *S. aureus* colonies recovered from infected animals showed patterns of sensitivity to AB-SA01 and its component phages that were similar to their respective parental strains, and no phage-resistant colonies were observed.

## 4. Discussion

The suitability of a medicinal product for human administration depends in part on the intrinsic characteristics of its active components. While no phage product to treat human infections has yet received market approval from the FDA or most of its global equivalents, the characteristics that make individual phages suitable for human use are commonly accepted within the phage research community [27,28,29,31] and generally supported by the FDA in public commentary on the subject [30]. AB-SA01, which is being developed to treat *S. aureus* infections, consists of three component phages that each meet these criteria in that they are: obligately lytic (not temperate), kill a wide range of clinical *S. aureus* strains, are incapable of specialized transduction and likely incapable of generalized transduction, and no bacterial virulence factors or drug resistance genes were identified by whole genome sequence analysis. Since a potential advantage of phage therapy is that it can be targeted to a pathogen of interest and therefore cause less disruption of the patient’s commensal flora than a broad-spectrum antibiotic, it is relevant that the AB-SA01 component phages appear to be specific to *Staphylococcus spp*., exhibiting no in vitro cross-genus activity.

In addition to the characteristics of individual phages, there is a rationale for the specific combination of phages that makes up AB-SA01. Within AB-SA01, phages Sa83, Sa87, and J-Sa36 each contribute different anti-*S. aureus* activity to AB-SA01; there is evidence of occasional synergy to kill otherwise non-susceptible *S. aureus* strains, the intrinsic frequency of resistance within populations of sensitive bacteria is low, and complementation is possible when resistance does develop.

The clinical utility of an antibacterial agent depends in large part on its spectrum of activity against target pathogens and non-target bacteria. The in vitro activity of AB-SA01 is high and the percentage of susceptible isolates is nearly identical on MDR and non-MDR *S. aureus* strains. This is similar to results from an external study that looked at two of the three AB-SA01 component phages and found no significant association between phage susceptibility and antibiotic resistance among 65 clinical *S. aureus* isolates [44]. The apparently lower activity of AB-SA01 on the VISA strains is difficult to interpret because this panel represents a diversity of vancomycin resistance determinants and not a diversity of *S. aureus* strain backgrounds. For *S. aureus* strains with a known multilocus sequence type there was no apparent association between genetic lineage and phage sensitivity, which is not unexpected given that the housekeeping genes on which bacterial strain typing systems are based are not expected to affect phage adsorption, replication, or lysis. It is possible for bacteria to become resistant to phages by a variety of mechanisms such as mutations in cell surface receptors and CRISPR, restriction-modification, or abortive infection systems [45]. The AB-SA01 component phages were partially chosen based on empirical evidence that the individual phages can complement resistance that may arise to another component. This is somewhat analogous to antibiotics that target multiple critical points in bacterial metabolism. The frequency of spontaneous resistance to AB-SA01 was measured as no greater than 3 × 10^−9^. This value is less frequent than for rifampicin [22] and approximately 10-fold higher than for daptomycin and linezolid [46,47], though it may be an overestimate since none of the counted colonies proved to be heritably resistant to AB-SA01. Unlike static small molecules, phages also have the potential to evolve in situ, adapting to local bacterial populations and undergoing antagonistic co-evolution to bypass newly developed resistance [48,49]. How this will play out clinically remains to be seen. In vitro, mutual adaptation often leads to long-term maintenance of both phage and bacterial populations [49], but patterns of in vitro and in vivo mutation have been shown to differ [48]. The collective global experience treating single patients, including with AB-SA01 [50], strongly suggests that phage administration can lead to clinical resolution of infection, sometimes with confirmed pathogen eradication [51,52,53,54,55,56,57,58,59,60].

The rare instances in which AB-SA01 formed plaques on a *S. aureus* strain, even though the individually tested component phages did not, are intriguing. Between-phage synergy has not been extensively studied. Commonly proposed mechanisms tend to focus on combinations of unrelated or distantly related phages in which, for example, two phages use different receptors [61] or one phage has a tailspike protein with depolymerase activity that degrades bacterial capsule and increases the accessibility of a cell surface receptor to a second phage that does possess such enzymatic activity [62]. This type of mechanism seems unlikely for AB-SA01 given the high degree of relatedness among its component phages. Our observations could conceivably be the result of interactions downstream of phage adsorption, e.g. an in trans effect in which each phage in a co-infected cell expresses gene(s) necessary for both phages to bypass an intracellular resistance mechanism that would otherwise have prevented the second phage from completing replication and lysis. However, this is hypothetical and would need to be investigated further.

A frequent point of discussion for phage therapy is whether a fixed composition phage product will remain active against globally circulating strains of bacteria for long enough to be useful. It has sometimes been postulated that the rapid pace of bacterial evolution might cause the clinical populations of a target pathogen to change rapidly enough that a phage product might no longer be relevant by the time it obtains market approval, or that once in use, resistance may develop too quickly for the phage product to remain useful. While resistance development is a relevant issue for any novel antibacterial, we are not aware of evidence that this risk or rate would be higher for phage products than for other antibacterial agents being developed with a similar focus on novel mechanisms of action and resistance management. On the contrary, traits such as complementation among component phages and phage evolution offer a means of combating this and the evolution of phage resistance often carries other fitness costs [63]. The data presented here show that, at least for *S. aureus*, it appears possible to create a fixed-composition phage product that has activity against the vast majority of circulating clinical strains over several years, including MDR strains. When looking only at in vitro AB-SA01 activity on the 2013, 2015, and 2016 Global Panels, it is possible to suppose that activity has been gradually decreasing over time. However, it is equally possible, especially considering the aggregate results shown in Table 1, that the three Global Panels represent a mean of approximately 94% with one result each above and below this percentage. Notably, 96.3% of the 27 contemporary *S. aureus* isolates received between 2017 and 2018 by AmpliPhi from physicians requesting AB-SA01 to treat individual patients with refractory *S. aureus* infections were sensitive to AB-SA01, offering “real-world” support for the expectation that AB-SA01 will be active against the isolates of patients not responding to antibiotics.

Murine models of acute pneumonia showed that AB-SA01 exhibits antibacterial activity in a vertebrate infection. Both the prototype product and AB-SA01 were as effective as vancomycin in reducing lung bacterial burdens. The efficacy of *S. aureus* phages was observed in both neutropenic and immunocompetent mice. In *Pseudomonas* *aeruginosa* pneumonia models, phages were observed to be ineffective in neutropenic mice even if the same phages had successfully controlled a similar infection in mice with different or no immune deficiencies [64]. This likely reflects a genuine difference between *S. aureus* and *P. aeruginosa* pathogenesis. Skerrett et al [65] reported that myeloid differentiation factor 88, which is required for neutrophil production, is essential for host defense against *P. aeruginosa* but not *S. aureus* pneumonia. Neutrophil elastase is important for eradication of *P. aeruginosa* by the host’s innate immune system [66], whereas *S. aureus* produces neutrophil elastase inhibitors and appears particularly resistant to neutrophil killing [67,68].

Most staphylococcal phages fall into three broad categories, temperate siphoviruses, obligately lytic myoviruses, and obligately lytic podoviruses [69]. The myoviruses have historically been grouped together and described as K-like or Twort-like [69], though recent taxonomic proposals divide them into four genera within a proposed *Twortvirinae* subfamily [70,71]. Collectively the staphylococcus myoviruses tend to have broad host ranges and are frequently discussed as actual and proposed components of therapeutic phage preparations [72,73]. Previous studies have also shown the potential of K-like *S. aureus* phages to treat biofilm-associated infections. Guimin et al. [44] studied two of the three AB-SA01 component phages and showed that they can reduce in vitro *S. aureus* biofilm. A four-phage mix containing the precursor of an AB-SA01 component phage also significantly degraded in vitro biofilm [74] and was used to treat mature *S. aureus* biofilm in a sheep sinus infection model [75]. After 3 days of treatment, the phage-treated sheep had significantly lower mucosal biofilm mass. Compared to the controls, the sheep were healthy, showed comparable levels of sinus mucosal inflammation and had healthy looking cilia.

Randomized, controlled clinical trials are needed to show that single-patient clinical observations and systematic preclinical data collected in a research environment will translate into broad clinical efficacy. The chemistry, manufacturing, and control aspects of AB-SA01 production (including but not limited to production, purification, quality control, storage, and stability) are beyond the scope of this manuscript. However, when added to the preclinical characterization data presented here, AB-SA01 and its associated data package enabled clinical studies under the oversight of the U.S. Food and Drug Administration and Australia’s Therapeutic Goods Administration (TGA). In 2016, the safety and tolerability of AB-SA01 was tested in two clinical trials: one healthy volunteer study in the United States under an Investigational New Drug (IND) application (NCT02757755) and one open-label investigator-initiated study in Australia among post-rhinoplasty CRS patients (ACTRN12616000002482). AB-SA01 was safe and well tolerated in both study populations. Among CRS patients, there were preliminary indications of efficacy that will need to be confirmed in placebo-controlled studies, such as reductions in sinus bacterial load, improved endoscopic findings, and general symptom improvement [76]. Finally, 15 patients with serious or life-threatening *S. aureus* infections not responding to antibiotics have received a cumulative total of more than 400 doses AB-SA01, including more than 300 administered intravenously under Individual Patient Expanded Access INDs in the United States or Australia’s Special Access Scheme. No serious adverse events attributed to AB-SA01 were reported and observations from these patients suggest that it may be fruitful to investigate the efficacy of AB-SA01 in randomized controlled trials involving indications such as bacteremia, native and prosthetic valve endocarditis, prosthetic joint infections, and ventricular assist device infections.

AB-SA01 is a well-characterized phage investigational product that has entered clinical development for the treatment of *S. aureus* infections. While it is frequently suggested that existing regulatory structures are not compatible with the clinical development of phage products or with the timely emergency treatment of patients not responding to antibiotics, AB-SA01 has thus far satisfied FDA and TGA requirements to conduct clinical trials and single-patient emergency treatment. As with any antibacterial, epidemiological shifts might eventually necessitate a compositional update. At that point, the accumulated clinical and regulatory experience that will hopefully have been established with fixed-composition products should pave the way for data-driven strategies to streamline updates to phage products.

## Figures and Tables

**Table 1:** In vitro antibacterial activity of AB-SA01 and its component phages on *Staphylococcus aureus*.

Panel Type		Phage	Percentage of Total Isolates Sensitive to Indicated Phage				% of MDR Isolates Sensitive to AB-SA01
	Panel		Sa83	Sa87	J-Sa36	AB-SA01	
Selection	AmpliPhi Reference Panel (*n* = 68) ^1^		85.2%	86.8%	76.4%	94.1%	94% (61/65)
Prevalence ^2^	2013 Global Panel (*n* = 53)		96.2%	96.2%	86.8%	100%	100% (38/38)
	2015 Global Panel (*n* = 60)		85.0%	93.3%	75.0%	96.7%	100% (28/28)
	2016 Global Panel (*n* = 60)		80.0%	83.3%	63.3%	88.3%	94% (30/32)
Targeted	CDC VISA Panel (*n* = 14)		64.3%	64.3%	64.3%	64.3%	69% (9/13)
	Regional USA300 Panel (*n* = 29) ^3^		100%	100%	100%	100%	100% (29/29)
	Ghent CRS Panel (*n* = 90)		NT	NT	NT	96.7%	Insufficient AST data
NA	Expanded Access Requests (*n* = 27) ^4^		85.2%	92.6%	88.9%	96.3%	Insufficient AST data
Summary Values			Diversity Panels: Selection and Prevalence (*n* = 241)			94.6%	-
			All Panels (*n* = 401)			94.5%	95% (*n* = 205)

Abbreviations: AST: Antibiotic Sensitivity Testing; CDC: Centers for Disease Control and Prevention; CRS: chronic rhinosinusitis; NA: not applicable; NT: not tested; MDR: multidrug resistant; VISA: vancomycin intermediate. ^1^ Includes all major hospital-acquired methicillin-resistant *S. aureus* (HA-MRSA) and community-acquired methicillin-resistant *S. aureus* (CA-MRSA) lineages. ^2^ Nearly random samples fitting geographic distribution 45% North America, 45% Europe., 10% Asia-Pacific, obtained from JMI Laboratories SENTRY program for antimicrobial surveillance. ^3^ Isolates selected from [40,41,42,43], each having a different Pulsed Field Gel Electrophoresis pattern. ^4^ Initial patient isolates submitted for sensitivity testing as part of requests for product use under U.S. Individual Patient Expanded Access or Australian Special Access Scheme policies between August 2017 and September 2018, inclusive. These policies allow patients with serious or life-threatening infections that are not responding to existing approved therapies to access investigational products on an emergency basis. AB-SA01 is listed as NCT03395769 for Expanded Access use in the United States.

**Table 2:** In vitro activity of AB-SA01 and its component phages on bacterial species other than *S. aureus*.

Bacteria		Number of Strains Tested	Number of Strains Productively Infected			
Order	Genus, Species		Sa83	Sa87	J-Sa36	AB-SA01
*Bacillales*	*Staphylococcus epidermidis*	5	2	2	2	2
*Lactobacillales*	*Streptococcus spp.*	3	0	0	0	0
*Corynebacteriales*	*Corynebacterium spp.*	4	0	0	0	0
*Micrococcales*	*Micrococcus luteus*	1	0	0	0	0
*Burkholderiales*	*Achromobacter xylosoxidans*	1	0	0	0	0
	*Burkholderia cepacia*	1	0	0	0	0
*Pseudomonales*	*Acinetobacter baumannii*	1	0	0	0	0
	*Pseudomonas aeruginosa*	3	0	0	0	0
	*Pseudomonas oryzihabitans*	1	0	0	0	0
*Enterobacteriales*	*Enterobacter cloacae*	1	0	0	0	0
	*Escherichia coli*	1	0	0	0	0
	*Klebsiella pneumoniae*	1	0	0	0	0
	*Pantoea agglomerans*	1	0	0	0	0
*Xanthamonadales*	*Stenotrophomonas maltophilia*	1	0	0	0	0

**Table 3:** Complementation among candidate phages.

Phage Used to Generate BIM	Bacterial Lawn	BIM Confirmation ^1^	Test for Complementation					
			Sa83	Sa87	J-Sa36	Sa76	Sa81	J-Sa37
Sa87	parental	S	S	S	S	S	S	S
	BIM 1	I	S	-	S	S	S	R
	BIM 2	I	S	-	S	S	S	R
	BIM 3	NG ^2^	-	-	-	-	-	-
	BIM 4	I	S	-	S	S	S	R
	BIM 5	I	S	-	I	S	S	R
	BIM 6	I	S	-	S	S	S	R
	BIM 7	I	S	-	S	S	S	R
	BIM 8	I	S	-	I	S	S	R
	BIM 9	I	S	-	I	S	S	R
	BIM 10	I	S	-	I	S	S	R
J-Sa36	Parental	S	S	S	S	-	-	S
	BIM 1	I	S	I	-	-	-	R
	BIM 2	I	I	I	-	-	-	R
	BIM 3	I	I	S	-	-	-	S
	BIM 4	I	I	I	-	-	-	S

^1^ Bacteriophage-insensitive mutant (BIM) confirmation conducted using same phage as in column 1. R (red): resistant (no phage activity seen within bacterial spot); I (yellow): intermediate (phage activity seen within bacterial spot); S (green): sensitive (<10 colonies within bacterial spot), -: not tested. ^2^ NG: no growth; BIM was not recovered during single colony purification and is therefore presumed to be sensitive; no other testing possible.

**Table 4:** Apparent frequency of intrinsic phage resistance in populations of *S. aureus* sensitive to AB-SA01 and its component phages.

Phage	After 24 h Plate Incubation			After 48 h Plate Incubation		
	Replicate 1 ^1^	Replicate 2	Replicate 3	Replicate 1	Replicate 2	Replicate 3
Sa83	1.1E-8	3.8E-9	5.0E-9	7.1E-9	3.8E-9	6.7E-9
Sa87	2.0E-8	5.0E-9	5.0E-9	1.7E-8	5.0E-9	5.0E-9
J-Sa36	2.9E-9	2.5E-9	1.2E-8	2.9E-9	1.3E-9	5.0E-9
AB-SA01 ^2^	1.4E-9	3.8E-9	3.3E-9	2.9E-9	0 ^3^	3.3E-9

^1^ Within each replicate, all aliquots of the same culture were exposed to each phage test sample. Since replicates contained a slightly different initial bacterial concentration, each replicate is displayed separately to allow for more accurate comparisons among the different phages. ^2^ Prepared as equal volume mixture of the three component stocks. ^3^ Limit of detection is 1.0E-9.

## Supplementary Materials

The following are available online at http://www.mdpi.com/1999-4915/11/1/88/s1, Table S1: Phage sensitivity of *S. aureus* strains with known MLST.
